# Analysis of Publication Productivity and Academic Rank of Ophthalmology Residency Program Directors in the United States

**DOI:** 10.7759/cureus.42989

**Published:** 2023-08-05

**Authors:** Deniz Oncel, Damla Oncel, Alara Kiliccioglu, Erin Sinai, Francis Arelleno, Banu Acikalin

**Affiliations:** 1 Ophthalmology, Loyola University Chicago Stritch School of Medicine, Chicago, USA; 2 Ophthalmology, Acibadem University School of Medicine, Istanbul, TUR; 3 Ophthalmology, Creighton University School of Medicine, Arizona, USA; 4 Ophthalmology, Georgetown University School of Medicine, Washington, DC, USA; 5 Ophthalmology, Fatih Sultan Mehmet Training and Research Hospital, University of Health Sciences, Istanbul, TUR

**Keywords:** gender comparison, residency program director, residency program, ophthalmology, gender disparities

## Abstract

Purpose: Female ophthalmologists are underrepresented in the field of ophthalmology. This study aimed to analyze the gender differences among ophthalmology residency program directors (PDs) in the United States with respect to academic rank, number of publications, and h-index.

Methods: This cross-sectional study evaluated 120 ophthalmology residency PDs from 120 ophthalmology residency programs during the 2022 San Francisco Match. The gender information was collected from institutional websites. The information regarding the state of each institute, academic rank, degree (MD or DO), age, and publication productivity was also recorded.

Results: From the 120 residency programs, 120 ophthalmology residency PDs were identified. Most PDs had an MD degree (118 out of 120, 98.3%), while only a few had a DO degree (2 out of 120, 1.7%). Only 31 (25.8%) out of 120 residency PDs were female. There was a statistically significant difference between female residency PDs and male residency PDs (p<0.0001). Male PDs had a higher h-index (15.2 ± 1.2) compared to their female counterparts (11.9 ± 0.97) (p=0.003). Regarding academic rank, male PD number was higher in each category, including assistant professor, associate professor, and full professor.

Conclusions: United States ophthalmology residency programs have a smaller portion of females compared to male PDs. Furthermore, full professors are more likely to be male, and males have higher publication productivity in terms of h-index. To promote equality among ophthalmologists, future initiatives should focus on addressing the gender disparities in ophthalmology residency programs and the selection of residency PDs.

## Introduction

In the United States, about 37.1% of physicians, 25.0% of ophthalmologists, and 29.6% of academic ophthalmology faculty are women [1,2]. There has been a rising number of women in the field of ophthalmology, and this trend is predicted to continue due to the fact that women now make up 50% of all medical graduates in the US and 44.3% of ophthalmology residents [1,2]. However, women still face underrepresentation not only in ophthalmology but also in other specialties such as urology and orthopedic surgery. Moreover, gender disparities persist in leadership positions on a national level [3,4]. Earlier research has indicated disparities in leadership roles, such as editor-in-chief positions and society presidents [4]. Tuli et al. found that the representation of genders in academic positions, such as associate professors, assistant professors, and full professors, has shown little change between 2003 and 2017 [4,5].

According to the Accreditation Council for Graduate Medical Education (ACGME), a residency program director (PD) is responsible for developing, overseeing, and improving the residency program they lead. They are accountable for the residents' personal and professional development, as well as the quality of patient care [6]. Being appointed as a residency PD in ophthalmology or any other medical field or specialty is considered a significant professional achievement. Although PDs play a crucial role in residency education in ophthalmology, there is a lack of comprehensive data on their demographic characteristics, publication productivity, and academic ranks. Recognizing the significance of this issue, the American Medical Association (AMA) has unveiled a plan to enhance diversity among physicians, and this initiative aims to investigate and address gender disparities in the medical field with a focus on improving the current situation [7]. Looking at gender differences among ophthalmologist residents' PDs will help the AMA accomplish its goal while also drawing awareness to the subject. This study aims to investigate the gender differences among ophthalmology residency PDs across the United States by analyzing the publication productivity and academic rank.

## Materials and methods

In this particular cross-sectional study, we investigated ophthalmology residency programs that were part of the 2022 San Francisco match in the United States. The data collection phase took place between January 2023 and April 2023, and we included a total of 120 programs without excluding any.

To gather information about the residency PDs, we accessed official institutional websites. We collected data on their academic rank, gender (female or male), publication productivity, degree (MD or DO), and h-indices. In cases where gender clarification was required, we employed various methods, such as visual identification through photographs, reviewing physician profiles, and utilizing additional online resources.

To assess scholarly activity, we searched the National Library of Medicine PubMed website using each faculty's first and last name, along with their middle initial when available. This allowed us to record the total number of peer-reviewed publications. To verify the accuracy of publication counts, we also consulted the Scopus database (Elsevier). The Scopus database provided information on the number of citations received by each author's papers, allowing us to calculate their h-index. The h-index is a measure of both productivity and impact, based on the highest number of publications that received at least the same number of citations [8]. For example, if a physician published 30 papers and their highest cited paper was cited 50 times, their h-index would be 30.

To ensure comprehensive data collection, we conducted online searches of the residency PDs' Scopus website profiles to identify any alternative names they might have used in the past. The Scopus database is a widely used resource containing a vast number of publication records from peer-reviewed sources, commonly employed in prior studies across various medical specialties [8-13].

To compare gender differences in scholarly productivity, publication count, and academic rank of the PDs, we utilized binary logistic regression models. Additionally, we computed descriptive statistics, including mean and standard deviation. The statistical comparisons were performed using SPSS 18 statistical software (SPSS version 18.0, IBM, Chicago, IL), with statistical significance set at p<0.05.

## Results

**Figure 1:**
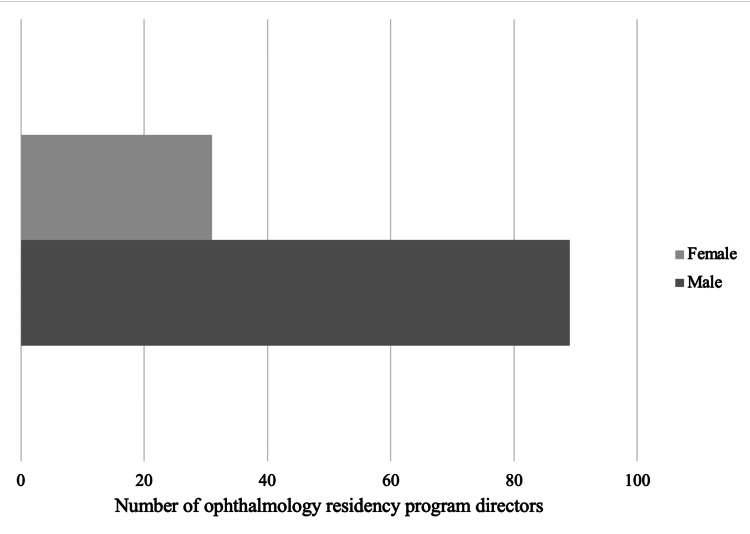
The comparison of gender (female or male) in ophthalmology residency program directors in the United States

A total of 120 residency PDs have been evaluated from 120 ophthalmology residency programs in the United States. The mean age of the PDs was 49 ± 10.9 years. Most of the PDs had an MD degree (118 out of 120, 98.3%), while only a few had a DO degree (2 out of 120, 1.7%). Thirty-one (25.8%) of the residency PDs were female, while 89 (74.2%) were male (Figure 1). When the male and female ophthalmologist numbers were compared, it resulted in a statistically significant difference (p<0.001).

Academic rank among ophthalmology residency PDs

**Figure 2:**
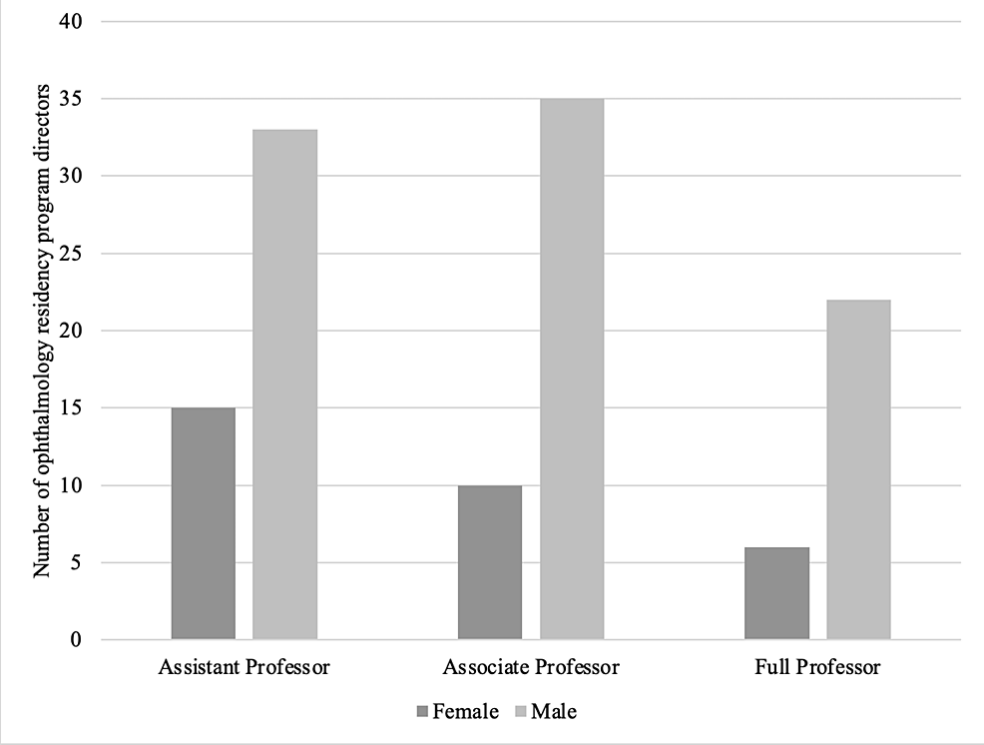
The comparison of gender (female or male) and academic rank of ophthalmology residency program directors in the United States

Among the 120 residency PDs analyzed, their academic ranks were distributed as follows: 48 (40.0%) were assistant professors, 45 (37.5%) were associate professors, and 28 (23.3%) were full professors. Among the assistant professors, 15 (31.3%) were female, while 33 (68.7%) were male. There were 10 (22.2%) female associate professors and 35 (77.8%) male associate professors. Regarding full professors, only six (21.4%) were female, while 22 (78.6%) were male. Male PDs were higher in number for each academic rank (Figure 2).

Publication productivity and h-index among ophthalmology residency PDs

**Figure 3:**
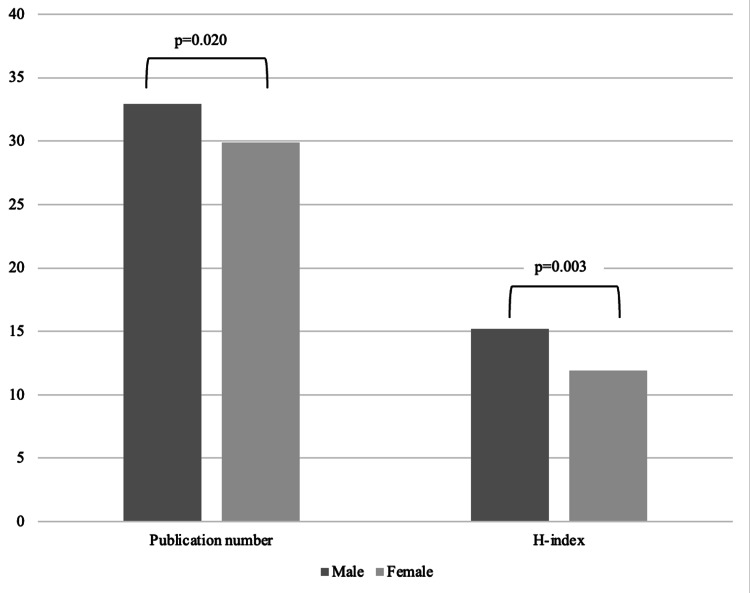
The comparison of gender (female or male) of ophthalmology residency program directors in the United States with relation to publication number and h-index

The average number of peer-reviewed publications among PDs was 35.3 ± 57.9. Female PDs had a mean of 29.9 ± 39.2 publications, while male PDs had an average of 32.9 ± 41.2 publications. However, there was no statistically significant difference in the mean publication number between male and female PDs (p=0.48) (Figure 3). When examining each academic rank separately, assistant professors had a mean of 5.4 ± 1.4 publications, while associate professors had a mean of 19.8 ± 14.2. Finally, full professors had a mean of 111.3 ± 82.6 publications. For each academic rank, there was no significant difference in publication productivity between male and female PDs (p=0.20).

We also examined the publication productivity of ophthalmology PDs in terms of their h-index, considering their gender. A higher h-index was found to be positively correlated with a higher academic rank, ranging from assistant professors to full professors (p<0.001). The mean h-index for each academic rank category was 4.0 ± 0.7 for assistant professors, 10.2 ± 1.2 for associate professors, and 28.0 ± 0.9 for full professors. In terms of gender differences, male PDs demonstrated a higher h-index (15.2 ± 1.2) compared to their female counterparts (11.9 ± 0.97) (p=0.003) (Figure 3). However, upon analyzing each academic rank separately, there were no significant differences in the h-indices between male and female PDs.

Gender differences in ophthalmology residency PDs among each state in the United States

When analyzed among each state in the United States, New York had the most residency programs. Of the 18 ophthalmology residency programs in New York, four (22.2%) programs included a female residency PD. California had the second most ophthalmology residency programs. Of the 10 ophthalmology residency programs, three (30.0%) included a female residency PD. Other states excluding the ones that had zero female residency program PDs, female residency PDs were 50% in Illinois, 100% in Indiana, 25% in Maryland, 100% in Rhode Island, 22.2% in Texas, 25% in Virginia, 50% in Washington, 50% in Wisconsin, 25% in Louisiana, 16.7% in Michigan, 50% in Missouri, 75% in North Carolina, 100% in Nebraska, 14.2% in Ohio, 100% in Connecticut.

## Discussion

Prior research has highlighted the issue of female underrepresentation in the field of ophthalmology [14,15]. Despite the increase in the number of women in medicine, females remain underrepresented in many specialties, particularly surgical fields [16,17]. Interestingly, there has been a 2.3% increase in the number of females in surgical specialties in recent years. However, in that same period, there has been a 2.5% decrease in the proportion of female ophthalmology residents [18]. The aim of the study was to investigate the gender differences among ophthalmology residency PDs in the United States while also analyzing the medical school degree obtained (MD or DO), the residency program state, academic rank, and publication productivity.

Overall, we have found a significantly smaller portion of female residency PDs compared to males in the field of ophthalmology. Thus, our analysis revealed a significant underrepresentation of female ophthalmology residency PDs. Specifically, only 25.8% of residency PD positions in ophthalmology are held by female PDs, indicating a need for more female leadership in this field. This percentage is comparable to the number of female PDs in other residency programs, including general surgery (29.8%) and internal medicine (29.9%) [19,20]. A cross-sectional study conducted in 2010 showed that 34% of the ophthalmology residency PDs were female, though they did not find a significant association between the gender of the residency PD and chairperson with the proportion of female faculty and residents [21]. In relation to this study conducted in 2010, our analysis shows a decrease in female PDs over the years, which is especially concerning considering the increased number of females in ophthalmology since 2010. Furthermore, when analyzed under academic rank, male PDs outnumbered female PDs in each category, assistant professor, associate professor, and full professor. In each academic rank category, we noted a significant underrepresentation of women (p<0.001). Our findings are consistent with previous studies showing that female faculty are disproportionately underrepresented in leadership positions within ophthalmology [2,15,22]. Another study by Lopez et al. also found a higher proportion of men in senior faculty positions than women [15]. Though there was no significant difference in publication amount between female and male PDs, there was a significant difference in h-index between female and male PDs.

This might be due to the fact that the h-index also takes into account the number of citations and the productivity of the publication amount each year, and the lack of women in leadership positions might lead to less attention on their publications. This discrepancy can also be caused by the unequal funding of research that women receive in comparison to men [23]. Previous studies have demonstrated that peer reviewers evaluating research grant proposals tend to overestimate male achievements and underestimate female accomplishments, highlighting a gender bias in the review process [24]. Another factor may be the gender bias in mentorship, with male mentors usually having male mentees. Women often receive less mentorship compared to men, and the lack of adequate mentorship can negatively impact women's career advancement and achievements [25,26]. Therefore, fewer leadership positions, less funding, and fewer mentors can all contribute to the lower h-indices of women in ophthalmology in their research publications. Females face greater challenges in balancing personal life, which includes taking care of their family, loved ones, household tasks, and professional lives, resulting in less extensive pursuit of promotions, publication productivity, or leadership roles as faculty compared to men [27].

Despite the rise in the number of women completing medical school, the field of ophthalmology continues to show a notable underrepresentation of females. Data from the AMA reveals that only 22.7% of ophthalmologists providing direct patient care are women, and a significant gender disparity exists in leadership roles, with 90% of current ophthalmology chairs being occupied by men [28]. Unfortunately, this gender imbalance is not unique to ophthalmology but extends to other specialties like orthopedic surgery, urology, plastic surgery, and otolaryngology, which also exhibit similar disparities. It is important to highlight that these specialties are known for their competitiveness, making it challenging for physicians, especially women, to secure positions in these fields.

The underrepresentation of women both in ophthalmology overall and in leadership positions is a concerning trend. This suggests that women may face obstacles in entering this competitive field and encounter even more difficulties in advancing to leadership roles. Previous research has shown a higher proportion of men serving in senior faculty positions compared to women [15], underscoring the persistence of the gender gap in academic ophthalmology and the limited presence of women in leadership roles. This disparity in leadership positions may further perpetuate the unequal cycle of inequality, possibly contributing to the ongoing underrepresentation of women in the field of ophthalmology.

Furthermore, our study also showed that the majority of PDs received an MD degree (98.3%). The low number of PDs that received a DO degree might be related to the fact that fewer osteopathic seniors match into ophthalmology residency programs. The 2022 Ophthalmology Residency Match Summary report showed that 76% of ophthalmology applicants were from a US allopathic medical school, while the remaining were from a US osteopathic medical school.

Our study encountered certain limitations that deserve attention. Notably, our reliance on online sources for data collection may have introduced inaccuracies, as we were unable to directly verify information with each department. Additionally, our use of the National Library of Medicine PubMed and Scopus databases to assess publication activity might not have captured all relevant data, especially when alternative names or variations were not easily identifiable. For example, identifying alternative names like maiden names presented challenges and could have affected our analysis of scholarly activity. Furthermore, gender assignment was based on visual assessment of photographs and names, which might not always align with an individual's self-identified gender. Despite these limitations, our study boasts several strengths. It represents the first analysis of residency PDs in the 2022 cycle, providing insights into the changing trends in the representation of women compared to men in this role. Moreover, we offer valuable information regarding demographics, medical school attended (DO or MD), the state of residency programs, and publication productivity, thus providing a comprehensive view of this subject matter.

In conclusion, ophthalmology residency programs exhibit a notable underrepresentation of females compared to males. To gain a more comprehensive understanding, it would be valuable to conduct a longitudinal analysis of gender disparities in residency PD positions, providing deeper insights and guidance for aspiring ophthalmologists. Moreover, future research should extend its scope to explore gender discrepancies among ophthalmologists in leadership roles within private clinics, hospital administration, and department chairs. By doing so, we can address and understand gender-related challenges across various facets of ophthalmology, facilitating progress toward greater equity and inclusion in the field.

## Conclusions

In conclusion, ophthalmology residency programs in the United States show a lower representation of female PDs compared to their male counterparts. Additionally, the position of full professor tends to be occupied more often by males, and males demonstrate higher publication productivity based on their h-index. Conducting longitudinal studies to analyze the trends of gender differences in residency PD positions could offer a more comprehensive approach and valuable insights for individuals considering a career in ophthalmology.

Moreover, future research should focus on examining gender disparities among ophthalmologists in leadership roles within private clinics, hospital administration, or department chairs. Investigating these areas will provide a deeper understanding of gender-related challenges and promote progress toward achieving greater gender equity and representation in ophthalmology.
